# Homotypic and heterotypic psychopathological continuity: a child cohort study

**DOI:** 10.1007/s00127-017-1396-7

**Published:** 2017-05-26

**Authors:** Mark Shevlin, Eoin McElroy, Jamie Murphy

**Affiliations:** School of Psychology and Psychology Research Institute, Ulster University, Magee campus, Londonderry, BT48 7JL Northern Ireland, UK

**Keywords:** Psychopathology, Comorbidity, Homotypic continuity, Heterotypic continuity, ALSPAC

## Abstract

**Background:**

Heterotypic psychopathological continuity (i.e. one disorder predicting another at a later time point) contradicts the conventional view that psychiatric disorders are discrete, static entities. Studying this phenomenon may help to tease out the complex mechanisms that underpin psychiatric comorbidity. To date, no studies have explicitly compared heterotypic effects within and across higher order dimensions of psychopathology.

**Methods:**

Patterns of homotypic and heterotypic psychopathological continuity were examined using cohort data from the Avon Longitudinal Study of Parents and Children (ALSPAC, *N* = 4815). Eight common psychiatric disorders were assessed at age 7.5 and again at age 14 years using the maternal report version of the Development and Well-Being Assessment (DAWBA). Cross-lagged models were used to compare patterns of homotypic and heterotypic continuity within and across three higher order dimensions of psychopathology; internalizing-fear, internalizing-distress, and externalizing.

**Results:**

Homotypic continuity was universal. Considerable heterotypic continuity was observed even after controlling for homotypic continuity and the presence of all disorders at baseline. Heterotypic continuity was more common within higher order dimensions, but a number of significant cross-dimension effects were observed, with ADHD acting as a strong predictor of subsequent internalizing disorders.

**Conclusions:**

Heterotypic continuity may reflect elements of shared aetiology, or local-level interactions between disorders.

## Introduction

A categorical nosology has formed the backbone of psychiatry since the publication of the third edition of the Diagnostic and Statistical Manual of Mental Disorders (DSM-III) [1]. This taxonomy conceptualises psychiatric disorders as dichotomous (i.e., either present or absent in an individual) and distinct entities. This paradigm has recently been challenged on a number of grounds, including the failure to identify reliable biomarkers for distinct diagnoses [2–6], the remarkable lack of predictive specificity for well-documented environmental risk factors [7–9], and the cross-disorder efficacy of psychopharmacological treatments [10]. Arguably, the most frequent criticism directed at categorical models concerns comorbidity. There is an abundance of epidemiological evidence which indicates that psychiatric disorders co-occur at greater than chance rates [11–13]. Furthermore, research suggests that psychiatric comorbidity is associated with greater overall psychopathological severity (e.g., distress, impaired functionality, and treatment need) [11, 13, 14].

Despite decades of research, our understanding of psychiatric comorbidity is modest at best. Cross-sectional studies have produced a number of transdiagnostic models of psychopathology which aim to describe this phenomenon. For example, the liability-spectrum model posits that psychopathology is better conceptualised as the continuous phenotypic expressions of a smaller number of naturally occurring broad [15]. Indeed, factor analytic studies have consistently identified higher order dimensions of psychopathology (e.g., internalizing and externalizing) that are proposed to account for comorbidity [15–19]. These dimensions mirror those originally proposed by Achenbach [20] in the field of child and adolescent psychiatry. Such models, however, have been plagued by notably high correlations between the factors themselves. More recently, these models have been expanded to include a general factor, labelled ‘*p*’, which is proposed to account for the co-occurrence of virtually all psychiatric disorders [21, 22]. Although it may be increasingly visible in the literature, a consistent interpretation of the *p*-factor has so far proven elusive [21, 23].

The above models are based on cross-sectional data and deal with comorbidity in terms of co-occurrence. An examination of psychiatric comorbidity in a longitudinal context (i.e. psychiatric continuity) may help to unpack the complex mechanisms that underpin comorbidity. Two types of continuity have been distinguished in the psychiatric literature: homotypic and heterotypic [13]. The term homotypic continuity is used when a particular psychiatric disorder predicts itself at a later time point [13, 24–26]. Conversely, heterotypic continuity occurs when a particular disorder predicts another disorder at a later time point [13, 24–26]. There is considerable evidence of both homotypic and heterotypic continuity between psychiatric disorders in both child/adolescent [26–32] and adult samples [24, 33]. Heterotypic continuity contradicts the all but abandoned argument that psychiatric disorders are distinct entities and may offer some insight into the development of psychiatric comorbidity.

Arguably the most comprehensive study of psychopathological continuity was conducted by Lahey et al. [24], who sought to examine whether heterotypic continuity was merely the result of uncontrolled homotypic continuity. In other words, they examined whether the longitudinal associations that were observed between psychiatric disorders could be attributed to shared aetiological influences which are purported to give rise to the cross-sectional correlations between psychopathological dimensions. Lahey et al. [24] hypothesised that, if the relative magnitudes of cross-sectional associations among different disorders at time 1 were of similar magnitudes to the heterotypic associations from time 1 to time 2, then this would indicate that shared aetiological influences (both biological and environmental) were driving both cross-sectional co-occurrence and longitudinal continuity. They claimed that a significant rank-order correlation between cross-sectional and heterotypic correlations, and greater heterotypic continuity within than across second-order domains would provide support for this hypothesis. They also stated that a failure to support this hypothesis would require either substantial modification or rejection of the liability-spectrum model.

Using data from the National Epidemiological Study on Alcohol and Related Conditions (NESARC; *N* = 28,958), Lahey et al. [24] examined the homotypic and heterotypic continuity of ten common psychiatric diagnoses, assessed 3 years apart. Tetrachoric correlations between all wave 1 and wave 2 diagnoses were estimated pairwise, each time controlling for the other 9 wave 1 diagnoses along with age and sex. Homotypic continuity was observed for each disorder. Heterotypic continuity was observed in almost all cases within three higher order factors of psychopathology (internalizing-fear, internalizing-distress, and externalizing). They also found less consistent, but still significant, heterotypic continuity across the second-order domains (e.g., disorders from distress predicting externalizing disorders at time 2). Furthermore, they found that the rank-order correlation between the cross-sectional and longitudinal associations was significant, indicating that the cross-sectional and heterotypic correlations were of a similar magnitude. They concluded that underlying genetic liabilities may predispose individuals to particular dimensions of psychopathology, whose manifestations change over time, possibly reflecting changes in environmental factors [24].

Further research of homotypic and heterotypic continuity is warranted if the hypothesis put forward by Lahey et al. [24] is to be substantiated. The present study aims to build on the previous work of Lahey et al. [24], which utilised an adult sample, by examining patterns of homotypic and heterotypic continuity in a child/adolescent sample. Psychopathological continuity during the transition to adolescence warrants particular attention, given the many biological, cognitive, and social changes that typically occur at this time. As such, many common psychiatric disorders tend to emerge in the early adolescence [11], and an examination of homotypic and heterotypic psychopathological continuity during this period may provide key insights into the development of psychiatric sequelae.

Furthermore, this study also aims to build on the previous work of Lahey et al. [24] by addressing a number of methodological issues. First, they conducted their analyses pairwise each time controlling for the 9 other wave 1 diagnoses along with age and sex, increasing the likelihood of a type I error. Second, Lahey et al. [24] claimed that heterotypic continuity was stronger within rather than across the higher order domains based on the significance and magnitudes of the observed correlations, thus supporting the liability-spectrum model. No formal statistical tests were conducted to substantiate this claim. The present study aimed to explicitly test this hypothesis using a model building approach to examine patterns of homotypic and heterotypic continuity amongst common psychiatric disorders in a large cohort assessed from childhood through adolescence (age approximately 7.5–14 years). It was predicted that heterotypic continuity would be widespread, and that the effects would be stronger within dimensions rather than across dimensions.

## Methods

### Sample

The current study utilised data from mother–child pairs from the Avon Longitudinal Study of Parents and Children (ALSPAC) [34, 35]. The ALSPAC is a prospective cohort study of children born in the English county of Avon between April 1st 1991 and December 31st 1992. The initial ALSPAC cohort consisted of 14,541 pregnancies, with 13,978 children alive at the 1 year time point. The sample is broadly representative of the overall population of children in the UK [34, 35]. The ALSPAC was conducted to examine how genetic and environmental factors combine to influence health and development. The ALSPAC involved a diverse range of follow-ups, with 68 data collection points between birth and 18 years [34, 35]. Data were collected using self-report postal questionnaires (completed by the study mothers and mother’s partners) and yearly clinics for the study children from the age of 7 years [34, 35]. Please note that the study website contains details of all the data that are available through a fully searchable data dictionary (http://www.bris.ac.uk/alspac/researchers/data-access/data-dictionary/). Ethical approval for the study was obtained from the ALSPAC Ethics and Law Committee and the Local Research Ethics Committees. Further detailed descriptions of the ALSPAC can be found elsewhere [34, 35].

### Measures

Psychopathology was measured using the Development and Well-Being Assessment (DAWBA) [36]. The DAWBA is a structured clinical interview designed to diagnose psychiatric disorders in 5–16 years old based on ICD-10 and DSM-IV criteria. It is divided into 14 sections based on symptom profiles [36]. It contains questions regarding the frequency, severity, longevity, and the impact of symptoms. It also contains open-ended questions for clinical review [36]. Research indicates that the DAWBA is both a valid and reliable measure of psychopathology in clinical and general population contexts [36–38]. Parent-report, postal questionnaire versions of the DAWBA were administered when the study children were aged approximately 7.5, and 14 years. The following disorders (i.e. those assessed at both time points) were included in the present analysis: specific phobia (SPP), social phobia (SOP), generalized anxiety (GAD), major depression (DEP), post-traumatic stress (PTSD), attention/activity problems (ADHD), oppositional/defiant behaviour (ODD), and conduct problems (CD). Official DAWBA diagnoses based on clinical review were only available at one time point (7.5 years). As there were no clinical diagnoses available at the 14 year time point, the following comprehensive and conservative recoding strategy was adopted using available information from the 7.5 and 14 year time points.

The DAWBA asks questions about core symptoms of these disorders. If the respondents endorse the requisite symptoms (based on DSM criteria), respondents are then asked to rate the child’s level of distress due to these symptoms (e.g. “kr381: Degree to which general anxieties upset child”) and several questions regarding burden/impaired functionality are then asked (e.g. “Degree to which worries interfered how well child gets on with respondent/rest of family in day-to-day life”). Responses were indicated on a 4-point Likert scale; 1 = ‘Not at all’, 2 = ‘Only a little’, 3 = ‘Quite a lot’, and 4 = ‘A great deal’. Based on the ALSPAC codebook, responses to the burden/impaired functionality questions can be summed to create a total burden score. To create quasi-diagnostic variables that closely mirror DSM-IV diagnoses, children were coded with a 1 if they endorsed the requisite symptoms and demonstrated significant distress (score of 3 or 4 on distress questions) or impaired functionality/burden (a score of +2 standard deviations above the mean on total burden variable). Otherwise, children were coded with a 0 (no symptoms, or significant distress/burden). For ODD, teacher complaint was used in place of distress, as distress does not reflect ICD-10/DSM-IV criteria for ODD. The DAWBA measure of conduct disorder differs significantly from the other symptom profiles, as distress and impaired functionality do not reflect ICD-10 and DSM-IV criteria for conduct disorder. Based on ALSPAC codebook guidelines, a binary variable named ‘any frequent/definite conduct problems’ was computed at the two assessment waves. For this variable, children were coded with a 1 if their parents reported that they definitely/frequently told lies for personal benefit, started fights, bullied/threatened others, stayed out later than allowed, stole, ran away from home, or played truant. All other children were coded as 0. The above recoding strategies were applied to the eight symptom profiles at the two assessment waves. This recoding process resulted in 8 binary quasi-diagnostic variables (1 = present, 0 = absent) at ages 7.5 and 14 years.

### Attrition

As the aim of the present study was to examine the predictive relationships between disorders over time (rather than prevalence), analyses were conducted on a sub-sample rather than an imputed data set. The base sample consisted of all children who had complete DAWBA data available at the 7.5 year assessment (*n* = 6617). Of the base sample, 1802 (i.e., lost to attrition) did not have complete data at age 14. As such, analyses were conducted on the final sample which consisted of those who had data present at both time points (*n* = 4815). Compared with those who had complete data, those lost to attrition were more likely to be male (Χ^2^ = 11.32, *df* = 1, *p* = 0.001), come from an ethnic background (Χ^2^ = 9.8, *df* = 1, *p* = 0.002), have a mother with lower qualifications (Χ^2^ = 139.93, *df* = 4, *p* < 0.001), and have an externalizing disorder at 7.5 years (Χ^2^ = 15.08, *df* = 1, *p* < 0.001). The presence of an internalizing disorder at 7.5 years did not impact attrition (Χ^2^ = 1.26, *df* = 1, *p* = 0.263).

### Statistical analysis

**Fig. 1:**
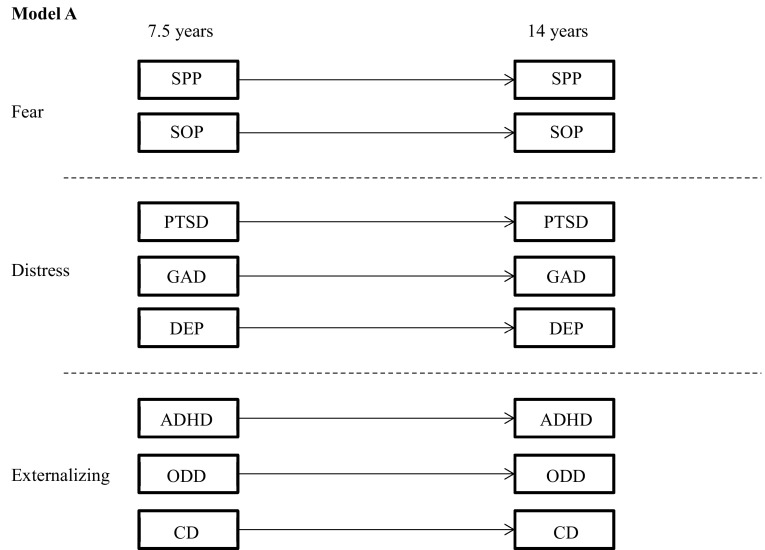
Model A. Homotypic continuity only. *SPP* specific phobia, *SOP* social phobia, *PTSD* post-traumatic stress disorder, *GAD* generalized anxiety, *DEP* major depression, *ADHD* attention/hyperactivity, *ODD* oppositional/defiant behaviour, *CD* conduct problems

**Fig. 2:**
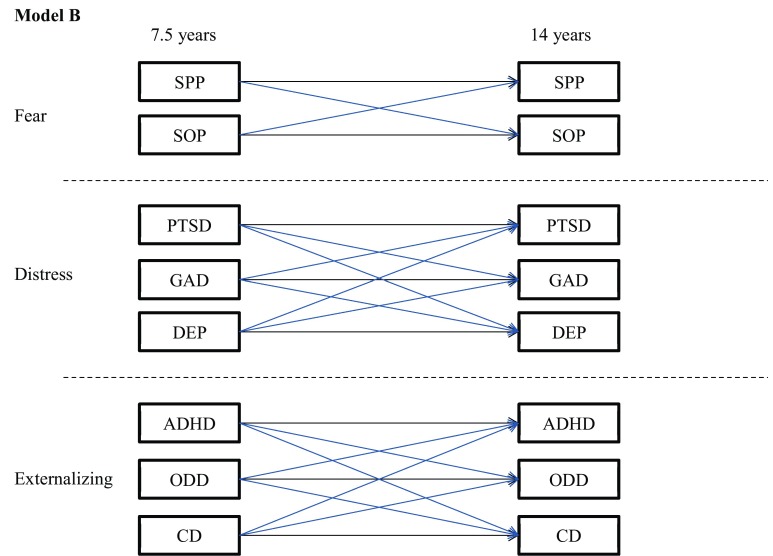
Model B. Homotypic continuity and heterotypic continuity within higher order dimensions only. *SPP* specific phobia, *SOP* social phobia, *PTSD* post-traumatic stress disorder, *GAD* generalized anxiety, *DEP* major depression, *ADHD* attention/hyperactivity, *ODD* oppositional/defiant behaviour, *CD* conduct problems

**Fig. 3:**
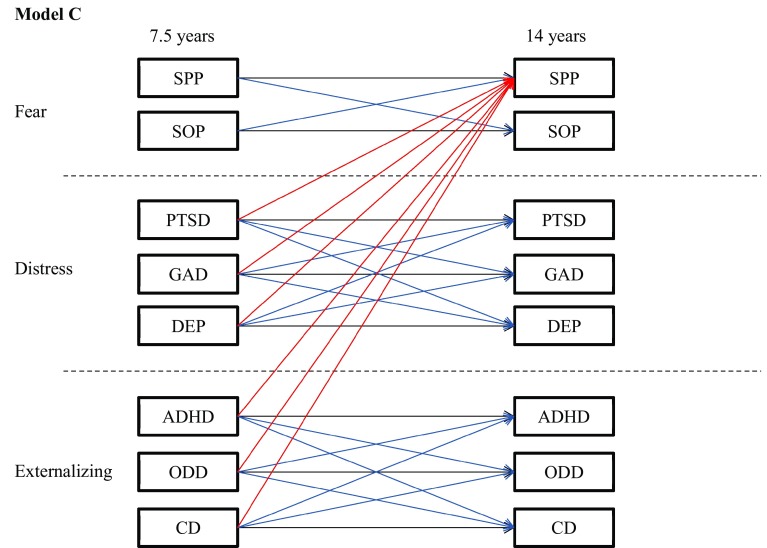
Model C. Homotypic continuity and heterotypic continuity within higher order dimensions only. For clarity, cross-dimension paths shown only for SPP. Actual model contained cross-dimensional paths for all outcome variables. *SPP* specific phobia, *SOP* social phobia, *PTSD* post-traumatic stress disorder, *GAD* generalized anxiety, *DEP* major depression, *ADHD* attention/hyperactivity, *ODD* oppositional/defiant behaviour, *CD* conduct problems

Bivariate tetrachoric correlations were computed between the diagnoses at age 7.5 and 14 years. To test the main hypothesis, a series of nested binary logistic regression models were specified and estimated. First, a model was tested in which the homotypic paths were freely estimate and heterotypic paths fixed at 0 (Fig. 1). Second, a model was tested in which both homotypic and heterotypic paths were freely estimated; however, the heterotypic paths were limited within the higher order dimensions of fear, distress, and externalizing only (Fig. 2). As per Lahey et al. [24], the disorders were partitioned into three higher order dimensions of internalizing-distress, internalizing-fear, and externalizing. Third, a model was tested in which all homotypic and heterotypic paths were freely estimated. In this model, each outcome variable was regressed on all disorders at the previous time point (Fig. 3). At each stage, the models were run under two conditions; (1) unadjusted for covariates and (2) adjusted for sex.

The analyses were conducted using Mplus v 7.0 [39], with robust maximum-likelihood estimation (MLR). The best fitting model was determined using the Akaike Information Criterion (AIC) [40], the Bayesian Information Criterion (BIC) [41], and the sample-size-adjusted Bayesian Information Criterion (ssaBIC) [42] with lower values indicative of better model fit.

## Results

Table 1 shows the frequencies and relative percentages of the disorders at the different time points. Comorbidity was high; 41% of those who screened positive for any disorder at age 7.5 screened positive for two or more disorder variables. At age 14, this figure was 43%. Table 2 shows the tetrachoric correlations between the disorders at age 7.5 and 14 years. All of the variables were significantly correlated, apart from SPP and CD at age 14 years. The largest correlation was between ADHD and ODD. Overall the correlations appeared larger within, rather than between, the specific dimensions.

**Table 1:** Frequencies and relative percentages quasi-diagnostic variables

		7.5 years	14 years
SPP	Present	383 (5.4%)	728 (11%)
	Absent	7012	6378
SOP	Present	330 (4.2%)	438 (6.6%)
	Absent	7819	6668
PTSD	Present	150 (1.9%)	240 (3.5%)
	Absent	7964	6866
GAD	Present	464 (6.4%)	948 (15%)
	Absent	7647	6158
DEP	Present	405 (5.5%)	827 (13%)
	Absent	7371	6279
ADHD	Present	468 (6.3%)	818 (13%)
	Absent	7371	6288
ODD	Present	385 (5.1%)	443 (6.6%)
	Absent	7622	6663
CD	Present	544 (7.1%)	424 (6.5%)
	Absent	7650	6486

*SPP* specific phobia, *SOP* social phobia, *PTSD* post-traumatic stress disorder, *GAD* generalized anxiety, *DEP* major depression, *ADHD* attention/hyperactivity, *ODD* oppositional/defiant behaviour, *CD* conduct problems

**Table 2:** Bivariate tetrachoric correlations (std. error) between disorders at age 7.5 years (top) and 14 years (bottom)

	SOP	PTSD	GAD	DEP	ADHD	ODD	CD
SPP	0.47 (0.04)	0.42 (0.05)	0.54 (0.03)	0.41 (0.04)	0.39 (0.04)	0.35 (0.04)	0.17 (0.04)
SOP		0.44 (0.05)	0.58 (0.03)	0.43 (0.04)	0.48 (0.03)	0.45 (0.04)	0.27 (0.04)
PTSD			0.57 (0.04)	0.48 (0.04)	0.45 (0.04)	0.50 (0.04)	0.36 (0.05)
GAD				0.74 (0.02)	0.58 (0.03)	0.56 (0.03)	0.34 (0.03)
DEP					0.56 (0.03)	0.55 (0.03)	0.34 (0.04)
ADHD						0.89 (0.01)	0.53 (0.03)
ODD							0.62 (0.03)
SPP	0.37 (0.03)	0.19 (0.04)	0.37 (0.03)	0.25 (0.03)	0.16 (0.03)	0.13 (0.04)	0.01 (0.04)
SOP		0.34 (0.04)	0.52 (0.02)	0.39 (0.03)	0.32 (0.03)	0.32 (0.04)	0.19 (0.04)
PTSD			0.47 (0.03)	0.48 (0.03)	0.33 (0.04)	0.42 (0.04)	0.37 (0.04)
GAD				0.63 (0.02)	0.38 (0.03)	0.43 (0.03)	0.34 (0.03)
DEP					0.40 (0.03)	0.49 (0.03)	0.40 (0.03)
ADHD						0.79 (0.02)	0.51 (0.03)
ODD							0.65 (0.03)

*SPP* specific phobia, *SOP* social phobia, *PTSD* post-traumatic stress disorder, *GAD* generalized anxiety, *DEP* major depression, *ADHD* attention/hyperactivity, *ODD* oppositional/defiant behaviour, *CD* conduct problems

### Model results

The fit statistics for the competing binary logistic regression models are presented in Table 3. Model B (heterotypic paths within higher order dimensions) provided a significant improvement over Model A (homotypic paths only), as evidenced primarily by lower BIC values. Inspection of the fit statistics, however, indicated that Model C (heterotypic continuity across higher order dimensions) did not represent an improvement over Model B. This would appear to suggest that, while there was evidence of heterotypic continuity, it only occurred within the higher order dimensions.

**Table 3:** Fit statistics for competing models

Model	Loglikelihood	Free parameters	AIC	BIC	ssaBIC
Model A^a^	−10,092.541	16	20,217.082	20,321.148	20,270.305
Model A^b^	−10,031.512	24	20,111.025	20,267.123	20,190.860
Model B^a^	−9978.862	30	20,017.724	20,212.847	20,117.518
Model B^b^	−9920.362	38	19,916.725	20,163.881	20,043.130
Model C^a^	−9874.696	72	19,893.393	20,361.689	20,132.898
Model C^b^	−9804.399	80	19,768.798	20,289.127	20,034.915
Model D^a^	−9899.694	39	19,877.389	20,131.049	20,007.121
Model D^b^	−9832.139	47	19,758.278	20,063.971	19,914.621

*Model A* homotypic continuity only, *Model B* heterotypic continuity within higher order dimensions only, *Model C* heterotypic continuity within and between higher order dimensions, *Model D* revised model in which non-significant cross-domain paths were removed

*AIC* Akaike information criterion, *BIC* Bayesian information criterion, *ssaBIC* sample-size-adjusted BIC

^a^No control variables

^b^Adjusted for sex

However, an inspection of the individual effects in Model C identified a number of statistically significant heterotypic paths across dimensions. As such, Model C was re-specified to include only the significant cross-dimensional heterotypic paths (Model D). Model D led to an improvement in fit over Model B and, as such, was accepted as the best fitting model. Each model was then adjusted for sex, with an identical pattern of fit emerging (i.e., Model D performing best).

The homotypic and heterotypic effects for the best fitting model (Model D) adjusted for sex are presented as odds ratios (95% confidence intervals) in Table 4. Overall, the ORs were largest for the homotypic effects, ranging from 2.14 (95% CI 1.48–3.10) for DEP to 8.02 (95% CI 5.57–11.53) for SOP.

**Table 4:**
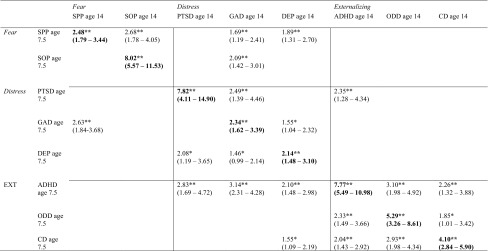
Odds ratios (95% confidence intervals) of time 2 disorders by time 1 disorders for the best fitting model (model D) adjusted for sex

Homotypic effects are in bold. Broken lines divide higher order dimensions. Significant effects only shown

*SPP* specific phobia, *SOP* social phobia, *PTSD* post-traumatic stress disorder, *GAD* generalized anxiety, *DEP* major depression, *ADHD* attention/hyperactivity, *ODD* oppositional/defiant behaviour, *CD* conduct problems

** *p* < 0.01; * *p* < 0.05

**Fig. 4:**
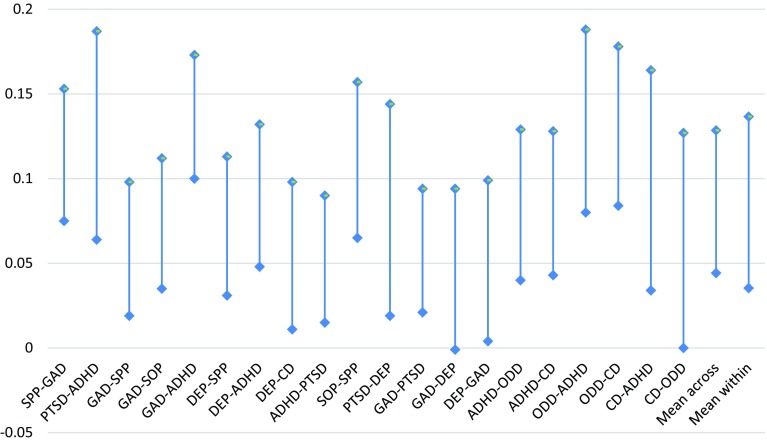
95% Confidence intervals of standardised effects. Dependent variables at age 14 listed first on *X-axis*

There was consistent heterotypic continuity within the externalizing dimension (i.e., each externalizing disorder at age 7.5 predicted all other externalizing disorders at age 14 years). This pattern was not observed for the fear or distress dimensions. There was also evidence of significant heterotypic continuity both within and across the broad higher order dimensions. ADHD demonstrated the most cross-domain effects, predicting PTSD, GAD, and DEP. Overall, the magnitude of these effects did not appear to be influenced by whether the two disorders in question were located within the same higher order dimension. To test the significance of the differences in effects within and across these domains, 95% confidence intervals for the standardised effects were plotted and inspected visually (Fig. 4). As per the guidelines of Cumming [43], an overlap of less than 50% was considered to reflect a statistically significant difference equivalent to *p* < 0.05, and confidence intervals that just touch were considered to reflect a significant difference at the *p* < 0.01 level. A number of individual effects differed significantly. For example, ADHD at age 7.5 was a stronger predictor of GAD at age 14 than any other disorder from the fear or distress dimensions. To compare the overall effect sizes for the within- and cross-domain effects, mean effect sizes and 95% CI values were calculated, and the results plotted (Fig. 4). There was complete overlap, suggesting no significant difference in the overall magnitude of effects for within- and cross-domain heterotypic continuity.

## Discussion

The present study sought to examine patterns of homotypic and heterotypic continuity amongst eight common psychiatric disorders in a large cohort assessed from childhood through adolescence (age approximately 7.5–14 years). Overall, it was predicted that heterotypic continuity (i.e., one disorder predicting a different disorder at a later time point) would be common. Out of 56 possible permutations of heterotypic continuity, 20 were statistically significant. This supported the hypothesis that heterotypic continuity would be common, even when controlling for homotypic continuity. This is consistent with a number of previous studies which have demonstrated widespread heterotypic continuity amongst psychiatric disorders [24, 25, 30]. Furthermore, the findings of the present study mirror those of Lahey et al. [24] by demonstrating that heterotypic continuity is not a result of uncontrolled homotypic continuity.

This study also aimed to expand upon the literature by directly comparing the heterotypic continuities of specific disorders within and between established higher order domains of psychopathology (fear, distress, and externalizing). To this end, a series of nested binary logistic regression models were estimated in which different degrees of heterotypic continuity were specified. It was predicted that, in line with hierarchical dimensional models of psychopathology, heterotypic effects would be stronger within than between these dimensions. Based on an inspection of the fit indices and effect sizes, it was concluded that a number of significant cross-dimension heterotypic effects warranted inclusion in the best fitting model. As such, it appears that heterotypic continuity does not occur solely within higher order dimensions of psychopathology. Furthermore, the overall magnitudes of the within-domain and cross-domain associations did not differ significantly.

### Interpreting heterotypic continuity

The analyses conducted in the present study, like those undertaken by Lahey et al. [24], were highly conservative. Each heterotypic effect identified was observed whilst controlling for homotypic continuity and the presence of all other disorders at baseline. As such, it appears that heterotypic continuity is not a statistical artefact, but rather a meaningful phenomenon. Interpreting this phenomenon, however, is far from straightforward. Ultimately, there are two opposing schools of thought; shared aetiology, and causal interaction. Lahey et al. [24] proposed a shared aetiological view of heterotypic continuity. They claimed that, as the cross-sectional and longitudinal heterotypic associations evidenced in their study were of a similar magnitude, this suggested that shared aetiological factors are responsible for these associations. In other words, the shared aetiological agents (e.g., genetic liabilities and environmental influences) that give rise to the cross-sectional associations also serve to drive the longitudinal associations. Such an interpretation is similar to that proposed by Caspi et al. [21], i.e., the *p*-factor model of psychopathology. In this model, the many heterotypic effects that exist between individual disorders are captured both by general and specific psychopathological factors, with *p* ultimately thought to reflect shared aetiology.

While such an interpretation is certainly plausible, it is far from confirmed. An alternative interpretation of heterotypic continuity can be found in the network approach to psychopathological comorbidity [44, 45]. This approach rejects the idea that the higher-order dimensions identified in cross-sectional research are solely the result of shared aetiological agents. It suggests that comorbidity is the result of complex networks of symptoms/disorders that directly and indirectly influence each other over time [44, 45]. As such, they suggest that *p* is a methodological artefact that is merely capturing a plethora of local-level interactions [46]. The idea that disorders may exert causal influences over each other is hardly new. Indeed, many developmental psychopathological models are based on this idea. One popular example is the “failure model”, which suggests that multiple disorder-level interactions may link externalizing behaviour (particularly attention deficit/hyperactivity problems) with subsequent internalizing problems [47, 48]. The “failure model” proposes that frequent or severe problems with attention may lead to negative responses from others (e.g., parents, teachers, and peers). These frequent negative responses may then lead to general distress within the child, which may eventually manifest as an internalizing disorder [49]. Although relatively speculative, the findings of the present study could be seen as supportive of this model, given that ADHD demonstrated the most frequent and strongest cross-domain heterotypic effects.

Given that experimental manipulation is not an option, it may be difficult if not impossible to definitively determine whether psychiatric comorbidity is the result of shared aetiological agents or networks of interactions. Studies of heterotypic continuity, however, may add support to one of the competing interpretations. For example, Lahey et al. [24] argued that, if the higher order domains of psychopathology are determined by shared risk, then heterotypic associations between disorders should be of greater magnitude within rather than across these domains. The present study, however, failed to observe such patterns of continuity. First, the fit indices suggested that the most parsimonious model included both within and cross-dimension paths. Second an inspection of the individual ORs indicated that the within and cross-dimension paths were generally of a similar magnitude, with ADHD demonstrating particularly strong effects both within and across dimensions. As such, there is little evidence in the present study to suggest that within-domain heterotypic continuity is stronger than between-domain heterotypic continuity.

The finding that within-domain and cross-domain heterotypic effects were of a similar magnitude is, perhaps, more in line with the network approach. The network approach to psychopathological comorbidity suggests that certain key symptoms or disorders serve as links between psychopathological domains (see ADHD example above). Under this assumption, it makes sense that cross-domain heterotypic effects would be as strong as within-domain effects. Ultimately, this interpretation remains highly speculative, and a significant replication of the present findings would be required to strengthen this argument.

It is worth noting that there is a growing acceptance that the difference between latent (i.e. common cause) and network approaches to the modelling of psychopathological data is philosophical rather than statistical [50]. As such, there may be different contexts to which each approach is more appropriate. For example, latent variable approaches to modelling may prove useful in identifying broad risk factors, e.g., genetic markers. The network approach may be better suited to the identification of key areas for intervention, such as symptoms/disorders that act as bridges in psychiatric comorbidity. Indeed, the present study serves as an example of this. The findings highlight the role of ADHD in the development of subsequent internalizing disorders. Indeed, clinicians may wish to consider comorbidity in a sequential sense, not just in a concurrent sense; the early intervention in cases of ADHD may prevent the subsequent development of comorbid internalizing problems.

### Limitations

The findings of the present study should be considered in light of the following limitations. First, it must be noted that the measured indicators were not clinical diagnoses, as no such data were available beyond the 7.5 year time point. To address this, however, a comprehensive and conservative recoding process was undertaken to capture distress and impaired functionality associated with the various psychiatric disorders at ages 7.5 and 14 years. Second, the present study was limited to eight common psychiatric disorders from two domains of psychopathology, internalizing and externalizing. Future research could include a broader range of disorders, along with disorders from other domains, e.g., psychotic disorders [18]. Third, as with all large-scale longitudinal studies, attrition was an issue. Although the sample size remained large, a significant amount of respondents were lost to attrition between the 7.5 and 14 years. Attrition was affected by demographic factors (sex, ethnicity, and maternal education). Studies have shown that selection bias due to demographic factors is unlikely to impact predictive relationships [51, 52]. Wolke et al. [51], however, demonstrated that selective dropout due to psychiatric variables (e.g., disruptive behaviour) had an impact on regression analyses in which psychiatric variables were the outcome, although such effects were marginal. In the present study, those with externalizing disorders at baseline were less likely to return for assessment at follow-up, which may have led to attenuated effects.

In conclusion, the present study examined patterns of homotypic and heterotypic continuity within the psychopathology of a cohort of children/adolescents aged 7.5–14 years. Both homotypic and heterotypic continuity were common, and heterotypic continuity was not explained by uncontrolled homotypic continuity. Although heterotypic continuity was more common within established higher order dimensions of psychopathology, a number of significant cross-domain effects were observed and were of a similar magnitude to the homotypic effects. It may be difficult to ascertain whether such effects are due to shared aetiological influences, or local-level interactions. Overall, these findings challenge the notion that psychiatric diagnoses reflect static and discrete entities.

## References

[1] American Psychiatric Association (1980). Diagnostic and statistical manual of mental disorders.

[2] Deacon BJ (2013). The biomedical model of mental disorder: a critical analysis of its validity, utility, and effects on psychotherapy research. Clin Psychol Rev.

[3] Kapur S, Phillips AG, Insel TR (2012). Why has it taken so long for biological psychiatry to develop clinical tests and what to do about it?. Mol Psychiatry.

[4] Boksa P (2013). A way forward for research on biomarkers for psychiatric disorders. J Psychiatry Neurosci.

[5] McLoughlin G, Makeig S, Tsuang MT (2014). In search of biomarkers in psychiatry: EEG-based measures of brain function. Am J Med Genet Part B Neuropsychiatr Genet.

[6] Cross-Disorder Group of the Psychiatric Genomics Consortium (2013). Identification of risk loci with shared effects on five major psychiatric disorders: a genome-wide analysis. Lancet.

[7] Green JG, McLaughlin KA, Berglund PA, Gruber MJ, Sampson NA, Zaslavsky AM, Kessler RC (2010). Childhood adversities and adult psychiatric disorders in the national comorbidity survey replication I: associations with first onset of DSM-IV disorders. Arch Gen Psychiatry.

[8] Kessler RC, McLaughlin KA, Green JG, Gruber MJ, Sampson NA, Zaslavsky AM, Benjet C (2010). Childhood adversities and adult psychopathology in the WHO World Mental Health Surveys. Br J Psychiatry.

[9] McElroy E, Shevlin M, Elklit A, Hyland P, Murphy S, Murphy J (2016). Prevalence and predictors of Axis I disorders in a large sample of treatment-seeking victims of sexual abuse and incest. Eur J Psychotraumatol.

[10] Stokes PE, Holtz A (1997). Fluoxetine tenth anniversary update: the progress continues. Clin Ther.

[11] Kessler RC, Chiu WT, Demler O, Walters EE (2005). Prevalence, severity, and comorbidity of 12-month DSM-IV disorders in the National Comorbidity Survey Replication. Arch Gen Psychiatry.

[12] Andrews G, Slade TIM, Issakidis C (2002). Deconstructing current comorbidity: data from the Australian National Survey of Mental Health and well-being. Br J Psychiatry.

[13] Angold A, Costello EJ, Erkanli A (1999). Comorbidity. J Child Psychol Psychiatry.

[14] Angst J, Sellaro R, Merikangas KR (2002). Multimorbidity of psychiatric disorders as an indicator of clinical severity. Eur Arch Psychiatry Clin Neurosci.

[15] Krueger RF, Markon KE (2006). Reinterpreting comorbidity: a model-based approach to understanding and classifying psychopathology. Annu Rev Clin Psychol.

[16] Wright AG, Krueger RF, Hobbs MJ, Markon KE, Eaton NR, Slade T (2013). The structure of psychopathology: toward an expanded quantitative empirical model. J Abnorm Psychol.

[17] Markon KE, Chmielewski M, Miller CJ (2011). The reliability and validity of discrete and continuous measures of psychopathology: a quantitative review. Psychol Bull.

[18] Kotov R, Chang SW, Fochtmann LJ, Mojtabai R, Carlson GA, Sedler MJ (2011). Schizophrenia in the internalizing-externalizing framework: a third dimension?. Schizophr Bull.

[19] Markon KE (2010). Modeling psychopathology structure: a symptom-level analysis of Axis I and II disorders. Psychol Med.

[20] Achenbach T (1966). The classification of children’s psychiatric symptoms: a factor-analytic study. Psychol Monogr Gener Appl.

[21] Caspi A, Houts R, Belsky D, Goldman-Mellor S, Harrington H, Israel S (2014). The *p* factor: one general psychopathology factor in the structure of psychiatric disorders?. Clin Psychol Sci.

[22] Lahey BB, Applegate B, Hakes JK, Zald DH, Hariri AR, Rathouz PJ (2012). Is there a general factor of prevalent psychopathology during adulthood?. J Abnorm Psychol.

[23] Greene AL, Eaton NR (2017). The temporal stability of the bifactor model of comorbidity: an examination of moderated continuity pathways. Compr Psychiatry.

[24] Lahey BB, Zald DH, Hakes JK, Krueger RF, Rathouz PJ (2014). Patterns of heterotypic continuity associated with the cross-sectional correlational structure of prevalent mental disorders in adults. JAMA Psychiatry.

[25] Reinke WM, Ostrander R (2008). Heterotyic and homotypic continuity: the moderating effects of age and gender. J Abnorm Child Psychol.

[26] Rutter M, Kim-Cohen J, Maughan B (2006). Continuities and discontinuities in psychopathology between childhood and adult life. Child Psychol Psychiatry.

[27] Ferdinand RF, Dieleman G, Ormel J, Verhulst FC (2007). Homotypic versus heterotypic continuity of anxiety symptoms in young adolescents: evidence for distinctions between DSM-IV subtypes. J Abnorm Child Psychol.

[28] Costello EJ, Mustillo S, Erkanli A, Keeler G, Angold A (2003). Prevalence and development of psychiatric disorders in childhood and adolescence. Arch Gen Psychiatry.

[29] Ormel J, Raven D, Oort FV, Hartman CA, Reijneveld SA, Veenstra R (2015). Mental health in Dutch adolescents: a TRAILS report on prevalence, severity, age of onset, continuity and co-morbidity of DSM disorders. Psychol Med.

[30] Kessler RC, Avenevoli S, McLaughlin KA, Green JG, Lakoma MD, Petukhova M (2012). Lifetime co-morbidity of DSM-IV disorders in the US national comorbidity survey replication adolescent supplement (NCS-A). Psychol Med.

[31] Bittner A, Egger HL, Erkanli A, Jane Costello E, Foley DL, Angold A (2007). What do childhood anxiety disorders predict?. J Child Psychol Psychiatry.

[32] Burke JD, Loeber R, Lahey BB, Rathouz PJ (2005). Developmental transitions among affective and behavioral disorders in adolescent boys. J Child Psychol Psychiatry.

[33] Flensborg-Madsen T, Knop J, Mortensen EL, Becker U, Sher L, Grønbæk M (2009). Alcohol use disorders increase the risk of completed suicide—irrespective of other psychiatric disorders. A longitudinal cohort study. Psychiatry Res.

[34] Boyd A, Golding J, Macleod J, Lawlor DA, Fraser A, Henderson J (2013). Cohort profile: the ‘children of the 90 s’—the index offspring of the Avon longitudinal study of parents and children. Int J Epidemiol.

[35] Fraser A, Macdonald-Wallis C, Tilling K, Boyd A, Golding J, Smith GD, Ring S (2013). Cohort profile: the Avon longitudinal study of parents and children: ALSPAC mothers cohort. Int J Epidemiol.

[36] Goodman R, Ford T, Richards H, Gatward R, Meltzer H (2000). The development and well-being assessment: description and initial validation of an integrated assessment of child and adolescent psychopathology. J Child Psychol Psychiatry.

[37] Brøndbo H, Mathiassen B, Martinussen M, Heiervang E, Eriksen M, Kvernmo S (2012). Agreement on web-based diagnoses and severity of mental health problems in Norwegian child and adolescent mental health services. Clin Pract Epidemiol Mental Health.

[38] Mullick MS, Goodman R (2005). The prevalence of psychiatric disorders among 5–10 year olds in rural, urban and slum areas in Bangladesh. Soc Psychiatry Psychiatr Epidemiol.

[39] Muthén BO, Muthén LK (2012). Mplus (version 7).

[40] Akaike H (1987). Factor analysis and AIC. Psychometrika.

[41] Schwarz G (1978). Estimating the dimension of a model. Ann Stat.

[42] Sclove SL (1987). Application of model-selection criteria to some problems in multivariate analysis. Psychometrika.

[43] Cumming G (2009). Inference by eye: reading the overlap of independent confidence intervals. Stat Med.

[44] Borsboom D, Cramer AO, Schmittmann VD, Epskamp S, Waldorp LJ (2011). The small world of psychopathology. PLoS One.

[45] Cramer A, Waldorp L, van der Maas H, Borsboom D (2010). Comorbidity: a network perspective. Behav Brain Sci.

[46] Murray AL, Eisner M, Ribeaud D (2016). The development of the general factor of psychopathology ‘p factor’ through childhood and adolescence. J Abnorm Child Psychol.

[47] Capaldi DM (1991). Co-occurrence of conduct problems and depressive symptoms in early adolescent boys: I. Familial factors and general adjustment at Grade 6. Dev Psychopathol.

[48] Capaldi DM (1992). Co-occurrence of conduct problems and depressive symptoms in early adolescent boys: II. A 2-year follow-up at Grade 8. Dev Psychopathol.

[49] Ostrander R, Herman KC (2006). Potential cognitive, parenting, and developmental mediators of the relationship between ADHD and depression. J Consult Clin Psychol.

[50] Beard C, Millner AJ, Forgeard MJC, Fried EI, Hsu KJ, Treadway MT, Björgvinsson T (2016). Network analysis of depression and anxiety symptom relationships in a psychiatric sample. Psychol Med.

[51] Wolke D, Waylen A, Samara M, Steer C, Goodman R, Ford T, Lamberts K (2009). Selective drop-out in longitudinal studies and non-biased prediction of behaviour disorders. Br J Psychiatry.

[52] Moffitt TE, Caspi A, Rutter M, Silva PA (2001). Sex differences in antisocial behaviour.

